# Supplementary material for: The purinergic receptor P2X7 and the NLRP3 inflammasome are druggable host factors required for SARS-CoV-2 infection
Source: Front Immunol. 2023 Oct 18;14:1270081. doi: 10.3389/fimmu.2023.1270081 (PMC10619763; doi:10.3389/fimmu.2023.1270081)
Supplement: Supplementary file 1 [file Presentation_1.pdf]

Supplementary material

The purinergic receptor P2X7 and the NLRP3 inflammasome are druggable host factors required for SARS-CoV-2 infection

Déborah Lécuyer, Roberta Nardacci, Désirée Tannous, Emie Gutierrez-Mateyron, Aurélia Deva Nathan, Frédéric Subra, Cristina Di Primio, Paola Quaranta, Vanessa Petit, Clémence Richetta, Ali Mostefa-Kara, Franca Del Nonno, Laura Falasca, Romain Marlin, Pauline Maisonnasse, Julia Delahousse, Juliette Pascaud, Eric Deprez, Marie Naigeon, Nathalie Chaput, Angelo Paci, Véronique Saada, David Ghez, Xavier Mariette, Mario Costa, Mauro Pistello, Awatef Allouch, Olivier Delelis, Mauro Piacentini, Roger Le Grand and Jean-Luc Perfettini*

*Correspondence to: jean-luc.perfettini@gustaveroussy.fr

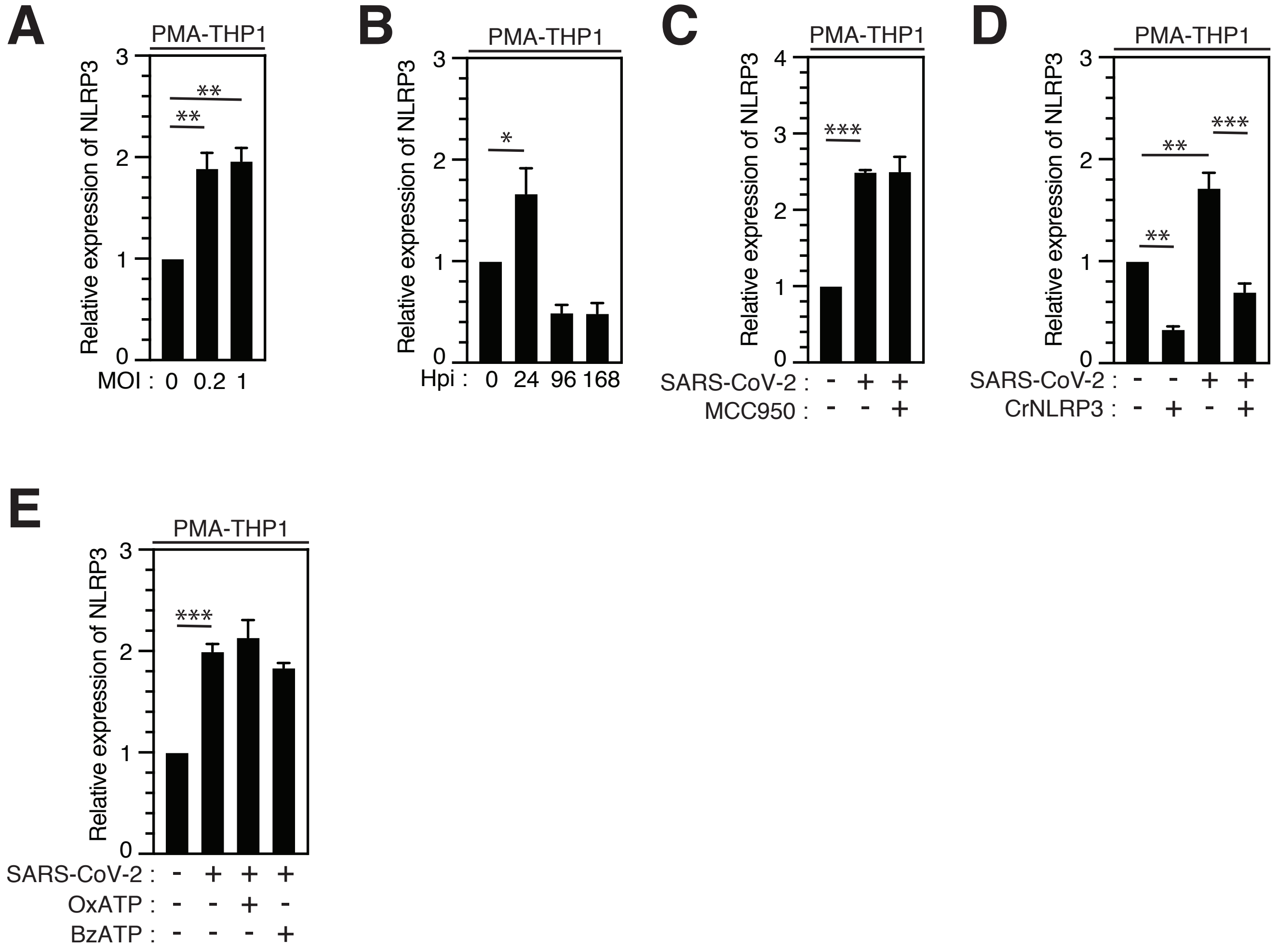
A
B
C
D
E
PMA-THP1
Relative expression of NLRP3
MOI : 0 0.2 1
Hpi : 0 24 96 168
SARS-CoV-2 : - + +
MCC950 : - - +
SARS-CoV-2 : - - + +
CrNLRP3 : - + - +
SARS-CoV-2 : - + + +
OxATP : - - + -
BzATP : - - - +

LECUYER#S1

Supplementary Figure 1 SARS-CoV-2 infection induces NLRP3 inflammasome activation in macrophages. A, B The relative S expression data from Figure 2A (**A**), Figure 2B (**B**), Figure 2G (**C**), Figure 2H (**D**) and Figure 2K (**E**) are presented as means ± SEM from at least 3 independent experiments. *p* values (**$p < 0.01$ and ***$p < 0.001$) were determined using one-way ANOVA Tukey's multiple comparisons test (**A-E**).

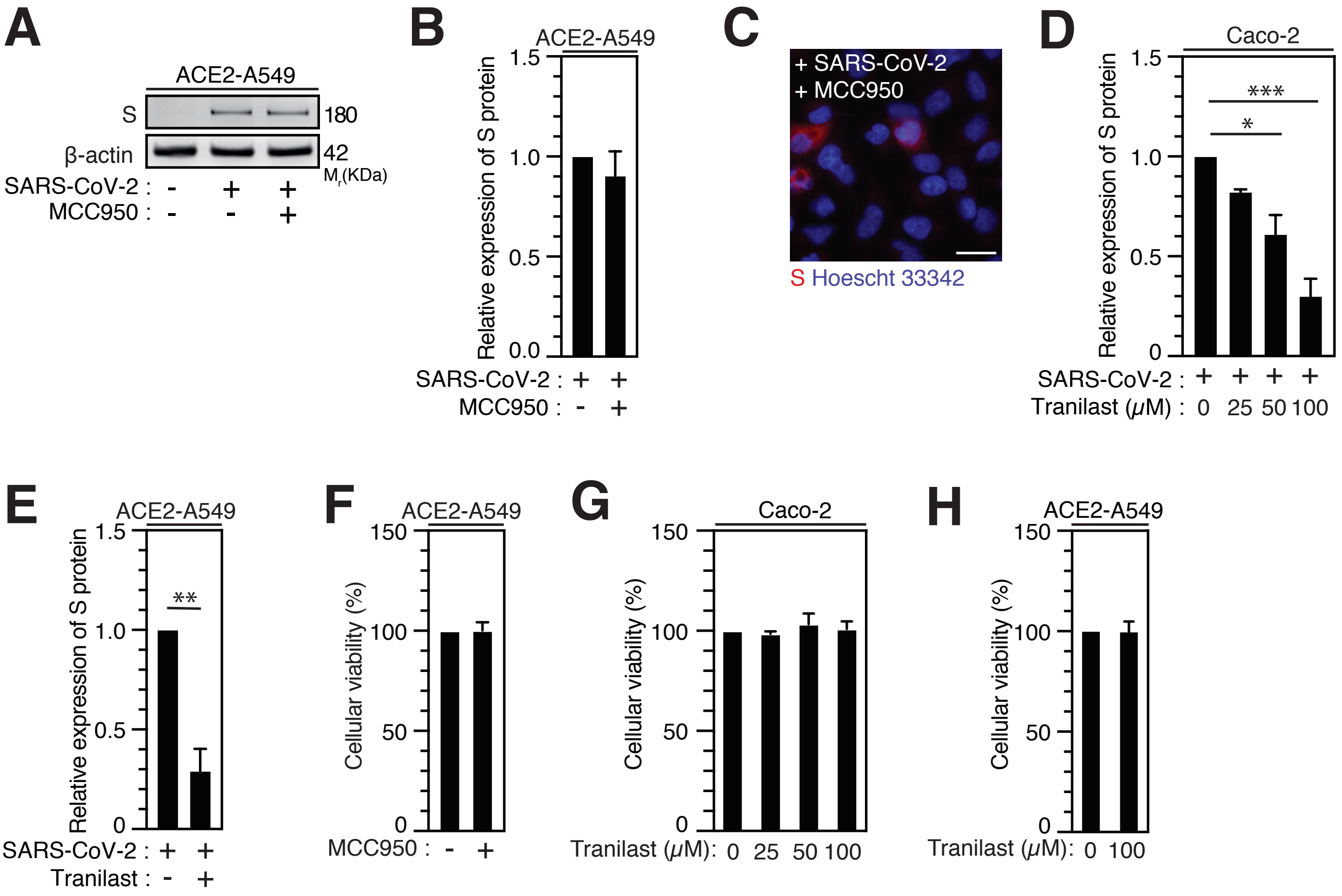

A
ACE2-A549
S
180
β-actin
42
Mr(KDa)
SARS-CoV-2 : - + +
MCC950 : - - +
B
ACE2-A549
Relative expression of S protein
1.5
1.0
0.5
0.0
SARS-CoV-2 : + +
MCC950 : - +
C
+ SARS-CoV-2
+ MCC950
S Hoescht 33342
D
Caco-2
Relative expression of S protein
1.5
1.0
0.5
0
***
*
SARS-CoV-2 : + + + +
Tranilast (μM) : 0 25 50 100
E
ACE2-A549
Relative expression of S protein
1.5
1.0
0.5
0
**
SARS-CoV-2 : + +
Tranilast : - +
F
ACE2-A549
Cellular viability (%)
150
100
50
0
MCC950 : - +
G
Caco-2
Cellular viability (%)
150
100
50
0
Tranilast (μM): 0 25 50 100
H
ACE2-A549
Cellular viability (%)
150
100
50
0
Tranilast (μM): 0 100

LECUYER#S2

Supplementary Figure 2 MCC950 and Tranilast exhibited distinct effects on SARS-CoV-2 replication and did not affect cellular viability. A-C ACE2-A549 cells were infected with SARS-CoV-2 at MOI = 2 for 48 hours with 20 µM MCC950 and evaluated for spike (S) and β-actin expressions by western bot (**A**, **B**) and spike (S) expression by fluorescence microscopy (**C**). Representative western blot (**A**), the relative S expression data from A (**B**) and representative image (**C**) are shown. Scale bar indicates 20 µm and DNA is detected using Hoechst 33342. **D, E** The relative S expression data from Figure 3F (**D**) and Figure 3G (**E**) are presented as means ± SEM from at least 3 independent experiments. **F-H** ACE2-A549 cells (**F, H**) or Caco-2 cells (**G**) were treated for 48 hours with 20 µM MCC950 (**F**) or indicated concentrations of Tranilast (**G, H**) and analyzed for viability using MTT assay. Percentages of viable cells are shown. Data are presented as means ± SEM from at least 3 independent experiments. *p* values (*$p < 0.05$, **$p < 0.01$ and ***$p < 0.001$) were determined using one-way ANOVA Tukey's multiple comparisons test (**D, G**) and unpaired t-test (**B**, **E**, **F** and **H**).

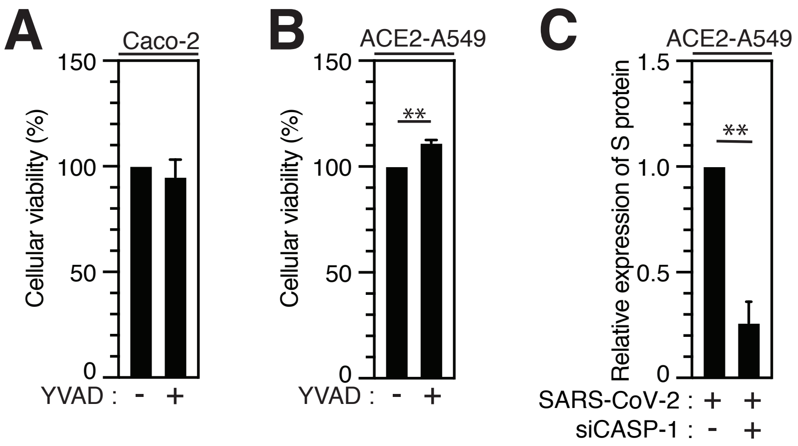
A
Caco-2
Cellular viability (%)
150
100
50
0
YVAD : - +
B
ACE2-A549
Cellular viability (%)
**
150
100
50
0
YVAD : - +
C
ACE2-A549
Relative expression of S protein
**
1.5
1.0
0.5
0
SARS-CoV-2 : + +
siCASP-1 : - +

LECUYER#S3

Supplementary Figure 3 Biological effects of YVAD and CASP-1 depletion on the viability of Caco-2 and ACE2-A549 cells or the relative expression of S protein. A, B Caco-2 cells (**A**) or ACE2-A549 cells (**B**) were treated for 48 hours with 100 μM YVAD and analyzed for viability using MTT assay. Percentages of viable cells are shown. **C** The relative S expression data from Figure 4H are presented as means ± SEM from at least 3 independent experiments. *p* values (**$p < 0.01$) were determined using unpaired t-test (**A-C**).

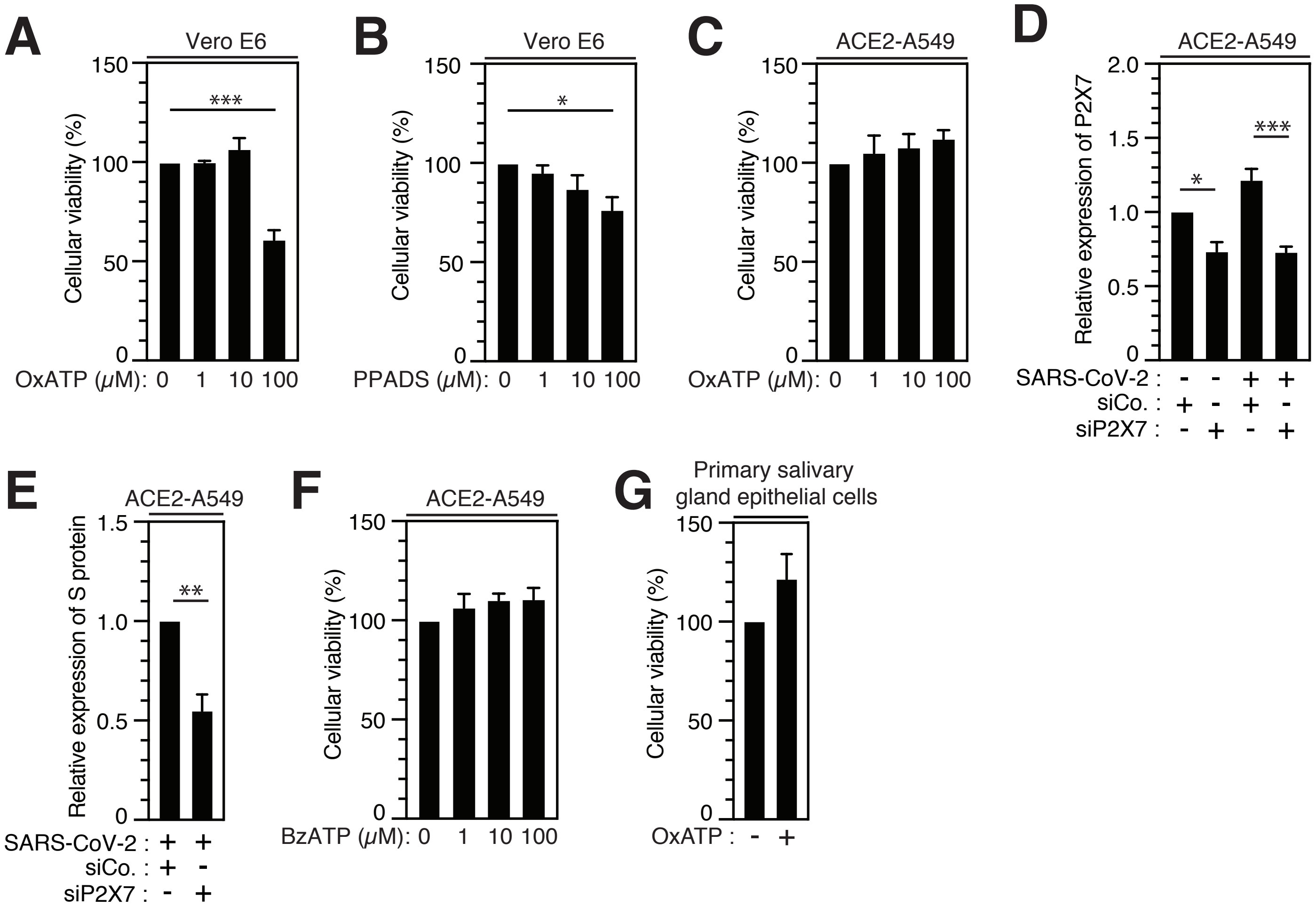
A
Vero E6
Cellular viability (%)
OxATP (μM): 0 1 10 100
B
Vero E6
PPADS (μM): 0 1 10 100
C
ACE2-A549
D
ACE2-A549
Relative expression of P2X7
SARS-CoV-2 :
siCo. :
siP2X7 :
E
ACE2-A549
Relative expression of S protein
F
ACE2-A549
BzATP (μM): 0 1 10 100
G
Primary salivary gland epithelial cells
OxATP :

LECUYER#S4

Supplementary Figure 4 Biological effects of OxATP, PPADS, BzATP or P2X7 depletion on the viability of Vero E6, ACE2-A549 and primary salivary gland epithelial cells or the relative expression of S protein. A-C Vero E6 (**A, B**) and ACE2-A549 cells (**C**) were treated for 24 hours (**A, B**) or 48 hours (**C**) with indicated concentrations of OxATP (**A, C**) or PPADS (**B**) and analyzed for viability using MTT assay. Percentages of viable cells are shown. **D, E** The relative expression data for P2X7 (**D**) and S protein (**E**) from Figure 5F are presented as means ± SEM from at least 3 independent experiments. **F** ACE2-A549 cells were treated for 48 hours with indicated concentrations of BzATP (**F**) and analyzed for viability using MTT assay. Percentages of viable cells are shown. **G** Primary salivary gland epithelial cells were treated for 48 hours with 100 μM of OxATP and analyzed for viability using MTT assay. Percentages of viable cells are shown. p values ($*p < 0.05$, $**p < 0.01$ and $***p < 0.001$) were determined using one-way ANOVA Tukey's multiple comparisons test (**A-D** and **F**) and unpaired t-test (**E, G**).

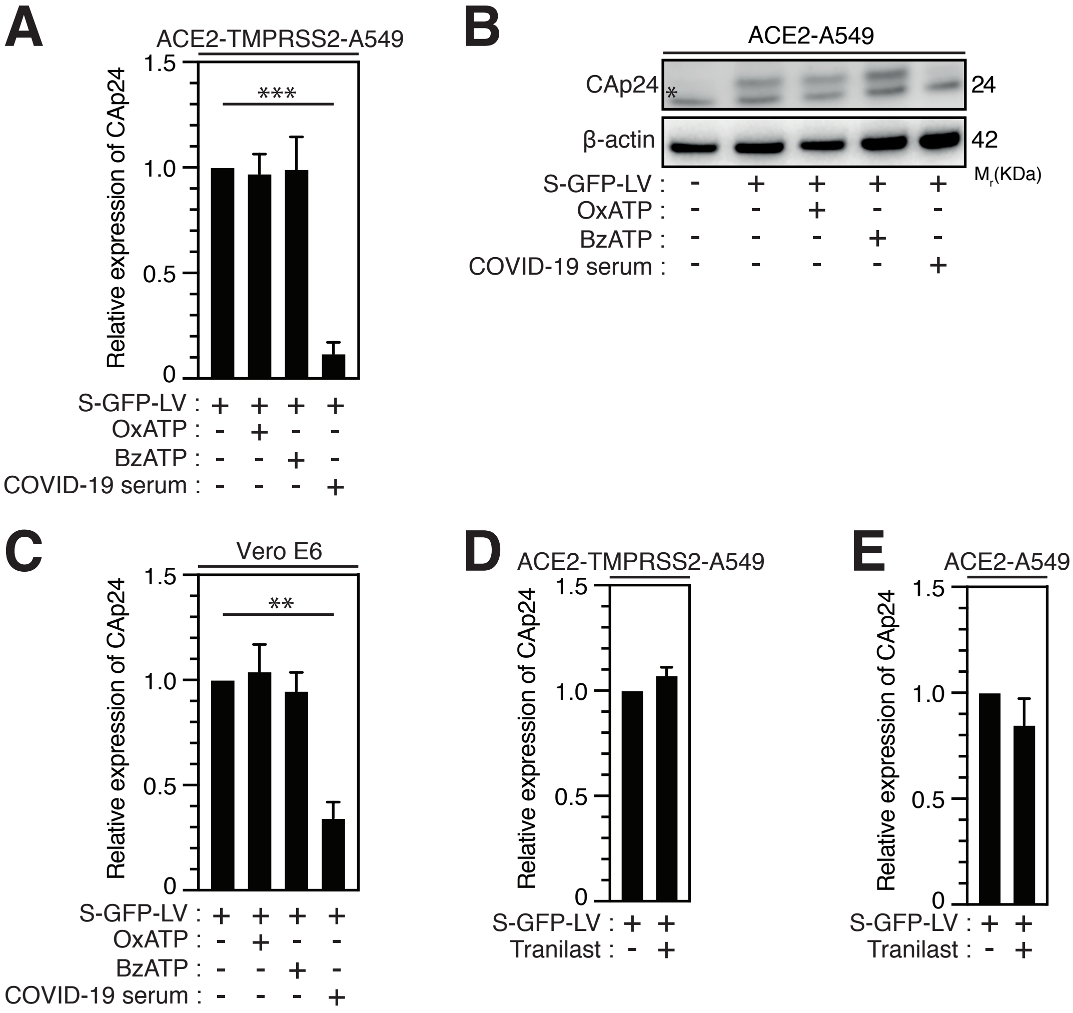
A
ACE2-TMPRSS2-A549
Relative expression of CAp24
1.5
1.0
0.5
0
***
S-GFP-LV : + + + +
OxATP : - + - -
BzATP : - - + -
COVID-19 serum : - - - +
B
ACE2-A549
CAp24
*
24
β-actin
42
Mr(KDa)
S-GFP-LV : - + + + +
OxATP : - - + - -
BzATP : - - - + -
COVID-19 serum : - - - - +
C
Vero E6
Relative expression of CAp24
1.5
1.0
0.5
0
**
S-GFP-LV : + + + +
OxATP : - + - -
BzATP : - - + -
COVID-19 serum : - - - +
D
ACE2-TMPRSS2-A549
Relative expression of CAp24
1.5
1.0
0.5
0
S-GFP-LV : + +
Tranilast : - +
E
ACE2-A549
Relative expression of CAp24
1.5
1.0
0.5
0
S-GFP-LV : + +
Tranilast : - +

LECUYER#S5

Supplementary Figure 5 P2X7 modulation did not affect SARS-CoV-2 entry in ACE2-A549 cells. A The relative CAp24 expression data from Figure 6G are presented as means ± SEM from at least 3 independent experiments. **B** ACE2-A549 cells were treated with 100 µM of OxATP or BzATP and infected with S-GFP-LV or with convalescent COVID-19 serum neutralized S-GFP-LV and analyzed for intracellular HIV-1 CAp24 capsid and β-actin expression. A western blot representative of two independent experiments is shown. The asterisk (*) is indicating a non-specific band. **C** The relative CAp24 expression data from Figure 6H are presented as means ± SEM from at least 3 independent experiments. **D** The relative CAp24 expression data from Figure 6O are presented as means ± SEM from at least 3 independent experiments. **E** The relative CAp24 expression data from Figure 6P are presented as means ± SEM from at least 3 independent experiments. *p* values (*$p < 0.05$, **$p < 0.01$ and ***$p < 0.001$) were determined using one-way ANOVA Tukey's multiple comparisons test (**A** and **C**) and unpaired t-test (**D, E**).

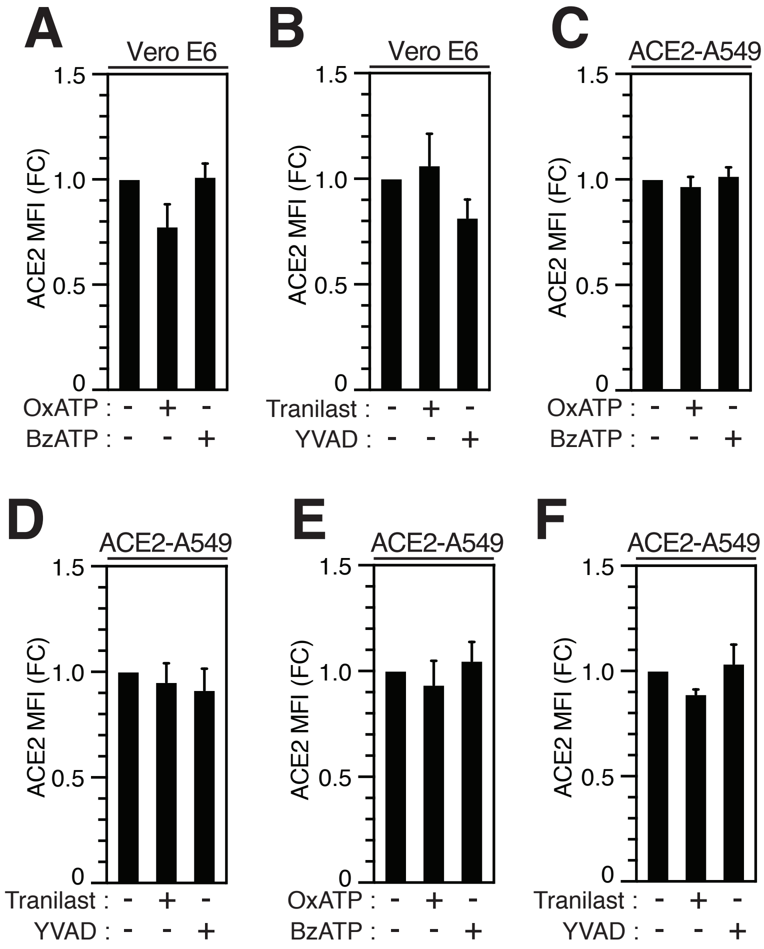

A
Vero E6
ACE2 MFI (FC)
1.5
1.0
0.5
0
OxATP : - + -
BzATP : - - +
B
Vero E6
ACE2 MFI (FC)
1.5
1.0
0.5
0
Tranilast : - + -
YVAD : - - +
C
ACE2-A549
ACE2 MFI (FC)
1.5
1.0
0.5
0
OxATP : - + -
BzATP : - - +
D
ACE2-A549
ACE2 MFI (FC)
1.5
1.0
0.5
0
Tranilast : - + -
YVAD : - - +
E
ACE2-A549
ACE2 MFI (FC)
1.5
1.0
0.5
0
OxATP : - + -
BzATP : - - +
F
ACE2-A549
ACE2 MFI (FC)
1.5
1.0
0.5
0
Tranilast : - + -
YVAD : - - +

LECUYER#S6

Supplementary Figure 6 Modulation of P2X7, NLRP3 inflammasome and caspase-1 biological activities did not affect membrane expression of ACE2. A-F Vero E6 (**A, B**) or ACE2-A549 (**C-F**) cells were treated for 6 hours (**A, B**), 4 hours (**C, D**) or 24 hours (**E, F**) with 10 μM OxATP (**A**), 100 μM OxATP (**C, E**), 100 μM BzATP (**A, C** and **E**), 100 μM Tranilast or 100 μM YVAD (**B, D** and **F**) and analyzed for ACE2 membrane expression by flow cytometry. Fold change (FC) are shown. Data are presented as means ± SEM from at least 3 independent experiments. No statistically differences were found using one-way ANOVA Tukey's multiple comparisons test (**A-F**).
